# Determinants of childhood immunization coverage in Somalia: evidence from the Somalia Demographic and Health Survey 2020

**DOI:** 10.1186/s13690-026-01924-0

**Published:** 2026-05-09

**Authors:** Sharmake Gaiye Bashir, Yakub Burhan Abdullahi, Yusuf Hared Abdi, Hiba Abdi Salad, Mohamed Sharif Abdi, Naima Ibrahim Ahmed, Nuradin Abdullahi Sheikh Rashid, Obasanjo Bolarinwa, Ahmed Abdinasir Abdulle

**Affiliations:** 1 Faculty of Health Science, Salaam University, Mogadishu, Somalia; 2 Center for Health Research and Innovation, Somali National University, Mogadishu, Somalia; 3 Department of Global Healthcare Management, York St John University, London, UK; 4 Demography and Population Studies Programme, University of the Witwatersrand, Johannesburg, South Africa

**Keywords:** Childhood immunization, Somalia, Vaccine coverage, Health equity, Socioeconomic determinants

## Abstract

**Background:**

Somalia has one of the lowest childhood immunization coverage rates globally, with only 34.8% of children aged 0–59 months having received at least one vaccine and a high burden of zero-dose children. Immunization uptake is influenced by socioeconomic, maternal, healthcare access, and geographic factors. This study examined determinants of childhood immunization coverage in Somalia to inform equity-focused strategies.

**Methods:**

A cross-sectional analysis was conducted using nationally representative data from the 2020 Somalia Demographic and Health Survey (SDHS), including 7,373 mother–child pairs. bivariate and multivariable logistic regression models assessed associations between sociodemographic, economic, maternal, healthcare access, and geographic characteristics and child vaccination status, accounting for survey design and confounders.

**Results:**

Overall vaccination coverage was 34.8%. Health facility delivery was the strongest independent predictor (AOR = 1.93; 95% CI:1.68–2.22; *p* < 0.001). Children from the highest household wealth quintile had higher odds than the poorest (AOR = 2.45; 95% CI:2.00–3.00; *p* < 0.001). Maternal primary and secondary education were positively associated with vaccination (AOR = 1.58; 95% CI:1.34–1.87 and AOR = 1.94; 95% CI:1.40–2.67; respectively; *p* < 0.001). Nomadic residence was associated with higher odds compared with rural residence (AOR = 1.69; 95% CI:1.46–1.96; *p* < 0.001). Compared with infants aged 0–11 months, children aged 12–23 months (AOR = 1.36; 95% CI:1.10–1.69; *p* = 0.005) and 24–59 months (AOR = 1.33; 95% CI:1.12–1.59; *p* = 0.001) were more likely to be vaccinated. Lack of radio exposure was associated with lower vaccination odds (AOR = 0.64; 95% CI:0.50–0.82; *p* < 0.001). Children living in Gedo region had markedly lower odds of vaccination than those in Awdal region (AOR = 0.26; 95% CI:0.17–0.39; *p* < 0.001).

**Conclusions:**

Childhood immunization coverage in Somalia remains critically low, reflecting socioeconomic, maternal, healthcare access, and geographic inequalities that require strategies targeting disadvantaged populations and regions.

**Table:** 

Text box 1. Contributions to the literature	
• Provides the first nationally representative evidence on childhood immunization determinants in Somalia using the 2020 DHS survey.	
• Highlights how place of delivery, maternal education, and household poverty shape childhood vaccination inequalities.	
• Shows that nomadic families may benefit from outreach strategies, challenging assumptions about hard-to-reach groups.	
• Demonstrates wide socio-economic and geographic disparities that require targeted policies and pro-poor investment.	
• Offers practical, context-specific insights for governments and partners to strengthen routine immunization in fragile and conflict-affected settings.	

## Introduction

Childhood immunization remains one of the most cost-effective public health interventions for reducing under-five mortality and morbidity from vaccine-preventable diseases globally [1, 2]. Since the establishment of the Expanded Programme on Immunization by the World Health Organization (WHO) in 1974, immunization efforts have saved an estimated 154 million lives over the past five decades [3, 4]. These achievements have been central to progress toward the United Nations Sustainable Development Goal 3.2, which aims to end all preventable deaths of newborns and children under five years of age by 2030, with target mortality rates of less than 25 per 1,000 live births [5, 6]. However, despite these remarkable gains, significant inequalities in immunization coverage persist between high- and low-income countries [7, 8]. Although high-income nations have achieved and maintained coverage levels exceeding 90%, many low- and lower-middle-income countries continue to struggle with suboptimal vaccination rates [9]. In 2023, an estimated 14.5 million children globally did not receive the first dose of diphtheria-tetanus-pertussis-containing vaccine, with 84% of these zero-dose children residing in low- and lower-middle-income countries [9]. This global immunization gap leaves millions of children vulnerable to life-threatening diseases and undermines the collective efforts to achieve universal health coverage and equitable access to life-saving interventions [6, 10].​.

Sub-Saharan Africa bears a disproportionate burden of incomplete childhood immunization and vaccine-preventable disease [11]. Despite considerable progress in expanding immunization programs across the region, vaccination coverage has stagnated at approximately 72% for the completion of the primary vaccine series, falling far short of the 90% target set by the Immunization Agenda 2030 [11]. Regional analyses revealed that full childhood immunization coverage among children aged 12–23 months ranges from 23.9% to 95.5% across different sub-Saharan African countries, highlighting substantial inter-country variations [12, 13]. These disparities are driven by multiple interconnected determinants, including maternal education, household socioeconomic status, place of residence, and access to health services [13, 14]. Evidence from demographic and health surveys across sub-Saharan Africa consistently demonstrates that children of mothers with secondary or higher education are significantly more likely to complete their immunization schedules than those whose mothers have no formal education [15]. Similarly, the household wealth quintile has emerged as a critical predictor of vaccination uptake, with children from the poorest households experiencing substantially lower coverage than their wealthier counterparts [16]. Geographic disparities between urban and rural areas further compounded these inequalities, as rural populations face barriers related to the distance to health facilities, limited cold-chain infrastructure, and inadequate vaccine supplies [17, 18].​.

Somalia represents one of the most challenging contexts for childhood immunization in sub-Saharan Africa and worldwide [19, 20]. The country’s health system has been severely weakened by more than three decades of armed conflict, political instability, recurrent droughts, and large-scale population displacement [21–23]. These protracted crises have resulted in fragmented health service delivery, destruction of health infrastructure, severe shortages of trained health workers, and disruption of vaccine supply chains and cold chain logistics [24]. The nomadic and semi-nomadic lifestyles of a significant proportion of Somalia’s population pose additional challenges for delivering routine immunization services through fixed health facilities [25]. Furthermore, insecurity in areas controlled by non-state-armed actors limits access to vulnerable populations and complicates the planning and implementation of immunization campaigns [20]. Against this backdrop, Somalia’s immunization coverage remains among the lowest in the world [19]. An analysis of the 2020 Somalia Demographic and Health Survey revealed that approximately 60.2% of children aged 12–23 months were zero-dose children who had not received any of the four basic routine vaccines [25]. Full immunization coverage in Somalia has been estimated to be only 20%–34%, with substantial regional disparities between urban, rural, and nomadic populations [26]. Children living in rural and nomadic areas were significantly more likely to be zero-dose than their urban counterparts [25]. Despite support from international partners, including Gavi, the Vaccine Alliance, WHO, and UNICEF, and the implementation of innovative vaccination delivery strategies such as integrated health camps in insecure areas, Somalia continues to face formidable obstacles in achieving equitable immunization coverage [20].​.

In Somalia, childhood immunization is delivered through the Expanded Programme on Immunization (EPI), which follows World Health Organization guidelines and targets protection against major vaccine-preventable diseases, including tuberculosis (BCG), diphtheria, tetanus, pertussis, poliomyelitis, Haemophilus influenzae type b (Hib), measles, and hepatitis B [20, 27]. The national immunization schedule targets children under two years of age, with vaccination initiated at birth (BCG), followed by scheduled doses at 6, 10, and 14 weeks for the pentavalent vaccine (DPT–HepB–Hib), and measles vaccination administered at 9 and 15 months [19, 28]. Childhood vaccination in Somalia is not legally mandatory [20, 27]. Immunization services are provided through a combination of routine services at fixed public health facilities, mobile outreach activities aimed at rural and nomadic populations, and periodic immunization campaigns implemented during national immunization days or outbreak responses [19, 20]. These delivery modalities operate within a highly heterogeneous context, contributing to substantial regional and population-level variation in immunization coverage across Somalia [20, 27].

While several studies have examined immunization coverage in Somalia, most have been limited by small sample sizes, sub-national geographic focus, or reliance on facility-based data from specific districts, such as Mogadishu [29, 30]. These localized studies, although valuable, do not provide nationally representative estimates of immunization determinants that can inform comprehensive policy interventions [25, 28]. Furthermore, many previous analyses have utilized outdated data that do not reflect recent humanitarian challenges, including the COVID-19 pandemic and intensified drought-related displacement [31]. The 2020 Somalia Demographic and Health Survey represents a critical advancement in generating robust, nationally representative data on child health indicators, including immunization coverage, across diverse population subgroups and geographic regions [25, 26, 28]. Leveraging this comprehensive dataset enables rigorous examination of the multifaceted individual-, household-, and community-level determinants of childhood immunization in the Somali context [25, 28].​.

This study aimed to identify and analyze the determinants of childhood immunization coverage among children aged 0–59 months in Somalia using data from the Somalia Demographic and Health Survey 2020. By examining sociodemographic, economic, maternal, healthcare access, and geographic factors associated with immunization uptake, this study seeks to generate evidence-based insights that can inform targeted interventions to improve vaccination coverage and reduce disparities. The findings will contribute to strengthening Somalia’s immunization program, enhancing the country’s progress toward achieving Sustainable Development Goal targets, and ultimately reducing preventable child morbidity and mortality in this fragile and conflict-affected setting.

## Methodology

### Study design and data source

This study employed a cross-sectional analytical design, using data from the most recent SDHS 2020. SDHS is a nationally representative household survey designed to provide reliable information on population health, maternal and child health, and reproductive indicators. The survey was conducted by the Somalia National Bureau of Statistics in collaboration with the Ministry of Health and international partners. It utilizes a two-stage stratified cluster sampling technique to ensure representativeness across all regions and population groups, including urban, rural, and nomadic communities.

In the first stage, enumeration areas were selected using a probability proportional to size. In the second stage, households were systematically sampled within each cluster. All women aged 15–49 years who were permanent residents or visitors to the selected households were eligible to participate. Data for this study were extracted from the most recent live-born child per woman in the five years preceding the survey, aged 0–59 months and living with the mother at the time of the interview, in accordance with standard DHS methodology. After excluding observations with missing data on key variables, 7,373 mother–child pairs were included in the descriptive and bivariate analyses.

### Study variables

#### Dependent variable

The dependent variable was childhood immunization status derived from the SDHS variable *H10 (ever vaccinated)*. This variable reflects whether a child has received any vaccination, regardless of the specific type of antigen. It was dichotomized as “1” for vaccinated and “0” for not vaccinated. Thus, the analysis captured overall child immunization coverage rather than the single-vaccine (e.g., measles-only) status.

#### Independent variables

Independent variables were selected based on theoretical relevance and previous literature and included maternal age (*Maternal age was measured at the time of the survey*,* consistent with the structure of the SDHS recode files. Although maternal age at childbirth may be more informative for some outcomes*,* this information was not available for all children and is acknowledged as a limitation.*), child age group, birth order, place of delivery, type of residence, maternal education level, household wealth quintile, frequency of radio listening, frequency of television watching, sex of the child, and region of residence.

Type of residence was obtained from SDHS variable V025, classifying households as urban, rural, or nomadic. In the Somalia DHS, *nomadic* populations refer to mobile pastoralist households characterized by seasonal movement and temporary dwellings, while *rural* and *urban* populations represent settled communities in non-urban and urban settings, respectively, as defined during the SDHS sampling process.

Household wealth quintile was measured using the DHS Wealth Index, constructed through principal components analysis of household assets and dwelling characteristics. Households were ranked and divided into five nationally representative quintiles (lowest to highest), reflecting relative socioeconomic status rather than income or consumption.

### Data management and statistical analysis

All statistical analyses were performed using Stata version 14. Prior to the analysis, the dataset was cleaned, and the variables were recoded and labelled for consistency and clarity. Descriptive statistics were computed to summarize the sociodemographic characteristics of the respondents. Pearson’s chi-square test was used to examine the association between each independent variable and the dependent variable. Variables with a p-value ≤ 0.05 in bivariate analysis were included in the multivariable model. Multicollinearity was checked before performing the final regression.

Bivariate logistic regression models were fitted to estimate the Crude Odds Ratios (cORs) with 95% confidence intervals (CIs) for the relationship between each explanatory variable and vaccination status. Subsequently, a multivariable logistic regression model was employed to estimate the Adjusted Odds Ratios (aORs) with corresponding 95% CIs, controlling for potential confounders. Robust standard errors were applied to account for the complex survey design. Statistical significance was set at a two-sided p-value of < 0.05. Sampling weights were applied in all analyses to adjust for clustering, stratification, and unequal probability of selection, thus ensuring nationally representative estimates.

## Results

Among 7,373 children, the overall vaccination coverage was 34.8%, with pronounced educational gradients evident as coverage increased from 31.2% among children of mothers with no formal education to 59.4% among those whose mothers had higher education. Wealth-related inequities were particularly stark, with vaccination rates ranging from 21.7% in the lowest wealth quintile to 54.3% in the highest quintile, representing a nearly two-fold difference. The place of delivery emerged as a critical determinant, with children born in health facilities demonstrating significantly higher vaccination coverage (55.2%) than those delivered at home (29.9%). Vaccination coverage was also higher among children of second or higher birth order (37.4%) compared with first-born children (33.0%). Geographic variations were also notable, with vaccination rates varying widely across regions from 10.2% in Gedo to 59.7% in Togdheer. Interestingly, children from nomadic settlements showed a relatively higher coverage rate (42.3%) compared to rural (30.9%) and urban (32.0%) residents, contrary to the typical patterns observed in other contexts. Media exposure, particularly television access at least once a week, was associated with substantially higher vaccination rates (64.0%), suggesting the potential role of health communication interventions, as shown in Table 1.

**Table 1:** Sociodemographic and obstetric characteristics of children aged 0–59 months by vaccination status, Somalia, 2020 Somalia Demographic and Health Survey (*n* = 7,373)

Variable	Category	Frequency (%)	Vaccinated, *n* (%)	Not vaccinated, *n* (%)
Mother’s age group	15–24 years	2,438 (33.1)	855 (35.1)	1,583 (64.9)
	25–34 years	3,507 (47.6)	1,251 (35.7)	2,256 (64.3)
	35–49 years	1,428 (19.4)	459 (32.1)	969 (67.9)
Mother’s education	No education	6,179 (83.8)	1,925 (31.2)	4,254 (68.8)
	Primary	915 (12.4)	471 (51.5)	444 (48.5)
	Secondary	215 (2.9)	131 (60.9)	84 (39.1)
	Higher	64 (0.9)	38 (59.4)	26 (40.6)
Wealth index	Lowest	1,783 (24.2)	386 (21.7)	1,397 (78.3)
	Second	1,744 (23.7)	400 (22.9)	1,344 (77.1)
	Middle	1,543 (20.9)	653 (42.3)	890 (57.7)
	Fourth	1,295 (17.6)	579 (44.7)	716 (55.3)
	Highest	1,008 (13.7)	547 (54.3)	461 (45.7)
Child sex	Male	3,832 (52.0)	1,353 (35.3)	2,479 (64.7)
	Female	3,541 (48.0)	1,212 (34.2)	2,329 (65.8)
Child age group	0–11 months	823 (11.2)	265 (32.2)	558 (67.8)
	12–23 months	901 (12.2)	338 (37.5)	563 (62.5)
	24–59 months	5,649 (76.6)	1,962 (34.7)	3,687 (65.3)
Place of delivery	Home delivery	5,942 (80.6)	1,775 (29.9)	4,167 (70.1)
	Health facility delivery	1,431 (19.4)	790 (55.2)	641 (44.8)
Residence (V025)	Rural	2,002 (27.2)	619 (30.9)	1,383 (69.1)
	Urban	3,181 (43.1)	1,019 (32.0)	2,162 (68.0)
	Nomadic	2,190 (29.7)	927 (42.3)	1,263 (57.7)
Radio exposure	≥ once/week	406 (5.5)	223 (54.9)	183 (45.1)
	< once/week	203 (2.8)	107 (52.7)	96 (47.3)
	Not at all	6,764 (91.7)	2,235 (33.1)	4,529 (66.9)
TV exposure	≥ once/week	445 (6.0)	285 (64.0)	160 (36.0)
	< once/week	165 (2.2)	85 (51.5)	80 (48.5)
	Not at all	6,763 (91.7)	2,195 (32.5)	4,568 (67.5)
Birth order group	1st birth	4,394 (59.6)	1,450 (33.0)	2,944 (67.0)
	≥ 2 births	2,979 (40.4)	1,115 (37.4)	1,864 (62.6)
Region	Awdal	239 (3.2)	85 (35.6)	154 (64.4)
	Woqooyi Galbeed	400 (5.4)	143 (35.8)	257 (64.2)
	Togdheer	325 (4.4)	194 (59.7)	131 (40.3)
	Sool	394 (5.3)	228 (57.9)	166 (42.1)
	Sanaag	510 (6.9)	231 (45.3)	279 (54.7)
	Bari	471 (6.4)	177 (37.6)	294 (62.4)
	Nugaal	498 (6.8)	195 (39.2)	303 (60.8)
	Mudug	506 (6.9)	233 (46.0)	273 (54.0)
	Galgaduud	467 (6.3)	139 (29.8)	328 (70.2)
	Hiraan	390 (5.3)	110 (28.2)	280 (71.8)
	Middle Shabelle	435 (5.9)	153 (35.2)	282 (64.8)
	Banadir	930 (12.6)	236 (25.4)	694 (74.6)
	Bay	195 (2.6)	82 (42.1)	113 (57.9)
	Bakool	543 (7.4)	122 (22.5)	421 (77.5)
	Gedo	510 (6.9)	52 (10.2)	458 (89.8)
	Lower Juba	560 (7.6)	185 (33.0)	375 (67.0)

**Fig. 1:**
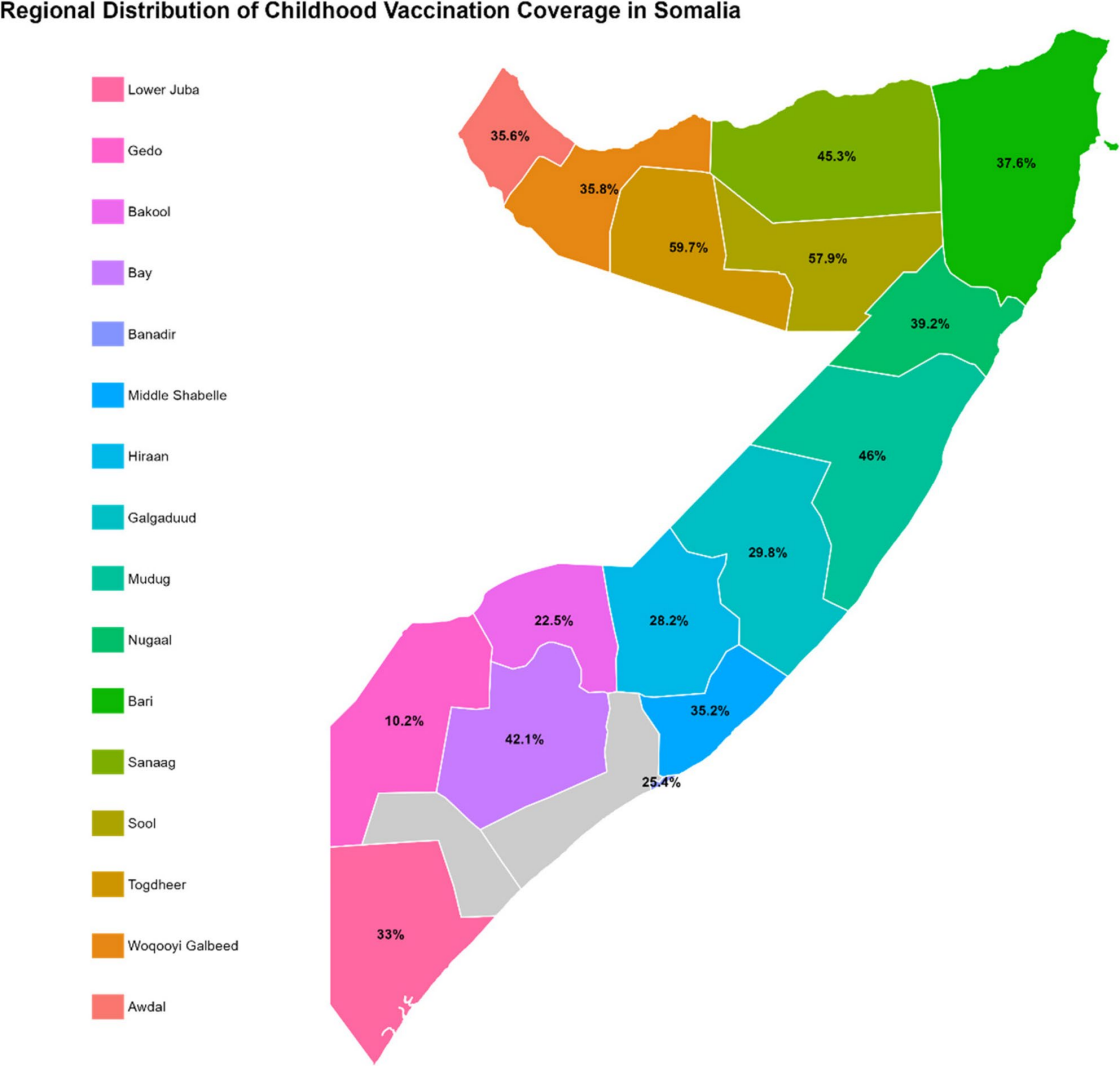
Regional disparities in childhood vaccination coverage (%) among children aged 0–59 months in Somalia, 2020 Somalia Demographic and Health Survey

Figure 1 illustrates substantial geographic heterogeneity in childhood vaccination coverage across Somalia. Vaccination coverage varied widely by region, ranging from as low as 10.2% in Gedo and 22.5% in Bakool to over 50% in Togdheer (59.7%) and Sool (57.9%). Moderate coverage levels were observed in regions such as Sanaag (45.3%), Mudug (46.0%), and Bay (42.1%), while lower coverage persisted in Banadir (25.4%), Hiraan (28.2%), and Galgaduud (29.8%). These spatial patterns highlight pronounced regional disparities in immunization uptake across the country.

The bivariate logistic regression analysis presented in Table 2 revealed several statistically significant determinants of childhood vaccination coverage in Somalia. Place of delivery emerged as the strongest predictor, with children born in health facilities demonstrating nearly threefold higher odds of vaccination than home-delivered children (cOR = 2.89, 95% CI: 2.57–3.26, *p* < 0.001), highlighting the critical role of facility-based delivery in facilitating vaccine uptake. Maternal education exhibited a strong dose-response relationship, with children of mothers with primary, secondary, or higher education showing progressively increasing odds of vaccination (COR = 2.34, 95% CI: 2.04–2.70; 3.45, 95% CI: 2.61–4.56; and 3.23, 95% CI: 1.96–5.33, respectively; all *p* < 0.001) compared with those whose mothers had no formal education. Household wealth demonstrated substantial socioeconomic gradients, as children from the highest wealth quintile had more than four times the odds of vaccination (COR = 4.29, 95% CI: 3.63–5.08, *p* < 0.001) relative to the poorest quintile, with the middle and fourth quintiles also showing significantly elevated odds (COR = 2.66, 95% CI: 2.28–3.09 and COR = 2.93, 95% CI: 2.50–3.42, respectively). Media exposure to both radio and television was strongly associated with vaccination status; children whose mothers watched television at least weekly had substantially higher vaccination odds, whereas lack of media exposure was associated with 73% lower odds (COR = 0.27, 95% CI: 0.22–0.33, *p* < 0.001). Regarding residence type, nomadic populations demonstrated 64% higher odds of vaccination (COR = 1.64, 95% CI: 1.44–1.86, *p* < 0.001) than rural residents, while urban residence showed no significant difference. Birth order was also significantly associated with vaccination status, with children of second or higher birth order having higher odds of vaccination compared with first-born children (COR = 1.21, 95% CI: 1.10–1.34, *p* < 0.001). Substantial regional variations were observed, with Togdheer (COR = 2.68, 95% CI: 1.90–3.79) and Sool (COR = 2.49, 95% CI: 1.79–3.47) demonstrating significantly higher vaccination odds, while the Gedo region showed markedly reduced odds (COR = 0.21, 95% CI: 0.14–0.30, *p* < 0.001) compared to the reference region Awdal. Notably, maternal age and child sex were not significantly associated with vaccination status in bivariate analysis.

**Table 2:** Bivariate logistic regression of sociodemographic and healthcare factors associated with childhood vaccination among children aged 0–59 months, Somalia, 2020 Somalia Demographic and Health Survey (*n* = 7,373)

Variable	Category	Vaccinated, *n* (%)	Not vaccinated, *n* (%)	COR (95% CI)	*p*-value
Mother’s age group	15–24 years	855 (33.1)	1,583 (32.9)	1	–
	25–34 years	1,251 (48.8)	2,256 (47.0)	1.06 (0.95–1.18)	0.295
	35–49 years	459 (18.1)	969 (20.1)	0.86 (0.75–1.00)	0.051
Child age group	0–11 months	265 (10.3)	558 (11.6)	1	–
	12–23 months	338 (13.2)	563 (11.8)	1.26 (1.04–1.54)	0.021
	24–59 months	1,962 (76.5)	3,687 (76.5)	1.12 (0.96–1.31)	0.153
Birth order	1st birth	1,450 (33.0)	2,944 (67.0)	1	—
	≥2 births	1,115 (37.4)	1,864 (62.6)	1.21 (1.10–1.34)	< 0.001
Place of delivery	Home delivery	1,775 (69.2)	4,167 (86.7)	1	–
	Health facility	790 (30.8)	641 (13.3)	2.89 (2.57–3.26)	< 0.001
Residence	Rural	619 (24.1)	1,383 (28.8)	1	–
	Urban	1,019 (39.7)	2,162 (45.0)	1.05 (0.93–1.19)	0.401
	Nomadic	927 (36.1)	1,263 (26.3)	1.64 (1.44–1.86)	< 0.001
Mother’s education	No education	1,925 (75.1)	4,254 (88.5)	1	–
	Primary	471 (18.4)	444 (9.3)	2.34 (2.04–2.70)	< 0.001
	Secondary	131 (5.1)	84 (1.7)	3.45 (2.61–4.56)	< 0.001
	Higher	38 (1.5)	26 (0.5)	3.23 (1.96–5.33)	< 0.001
Radio exposure	≥ once/week	223 (8.7)	183 (3.8)	1	–
	< once/week	107 (4.2)	96 (2.0)	0.91 (0.65–1.28)	0.605
	Not at all	2,235 (87.1)	4,529 (94.2)	0.40 (0.33–0.50)	< 0.001
TV exposure	≥ once/week	285 (11.1)	160 (3.3)	1	–
	< once/week	85 (3.3)	80 (1.6)	0.60 (0.42–0.86)	0.005
	Not at all	2,195 (85.6)	4,568 (95.0)	0.27 (0.22–0.33)	< 0.001
Wealth index	Lowest	386 (15.0)	1,397 (29.1)	1	–
	Second	400 (15.6)	1,344 (28.0)	1.08 (0.92–1.26)	0.359
	Middle	653 (25.5)	890 (18.5)	2.66 (2.28–3.09)	< 0.001
	Fourth	579 (22.6)	716 (14.9)	2.93 (2.50–3.42)	< 0.001
	Highest	547 (21.3)	461 (9.6)	4.29 (3.63–5.08)	< 0.001
Child sex	Male	1,353 (52.8)	2,479 (51.6)	1	–
	Female	1,212 (47.2)	2,329 (48.4)	0.95 (0.87–1.05)	0.331
Region	Awdal	85 (3.3)	154 (3.2)	1	–
	Woqooyi Galbeed	143 (5.6)	257 (5.4)	1.01 (0.72–1.41)	0.962
	Togdheer	194 (7.6)	131 (2.7)	2.68 (1.90–3.79)	< 0.001
	Sool	228 (8.9)	166 (3.5)	2.49 (1.79–3.47)	< 0.001
	Sanaag	231 (9.0)	279 (5.8)	1.50 (1.09–2.06)	0.012
	Bari	177 (6.9)	294 (6.1)	1.09 (0.79–1.51)	0.599
	Nugaal	195 (7.6)	303 (6.3)	1.17 (0.85–1.61)	0.347
	Mudug	233 (9.1)	273 (5.7)	1.55 (1.13–2.12)	0.007
	Galgaduud	139 (5.4)	328 (6.8)	0.77 (0.55–1.07)	0.118
	Hiraan	110 (4.3)	280 (5.8)	0.71 (0.50–1.00)	0.053
	Middle Shabelle	153 (5.9)	282 (5.9)	0.98 (0.71–1.37)	0.919
	Banadir	236 (9.2)	694 (14.4)	0.62 (0.45–0.83)	0.002
	Bay	82 (3.2)	113 (2.4)	1.31 (0.89–1.94)	0.168
	Bakool	122 (4.8)	421 (8.8)	0.53 (0.38–0.73)	< 0.001
	Gedo	52 (2.0)	458 (9.5)	0.21 (0.14–0.30)	< 0.001
	Lower Juba	185 (7.2)	375 (7.8)	0.89 (0.65–1.23)	0.489

The multivariable logistic regression analysis presented in Table 3 identifies independent determinants of childhood vaccination after adjusting for potential confounders, revealing nuanced patterns that differ from those observed in bivariate analysis. Place of delivery remained the most robust predictor, with health facility births associated with nearly double the odds of vaccination (AOR = 1.93, 95% CI: 1.68–2.22, *p* < 0.001) compared with home deliveries, underscoring the critical importance of facility-based care in vaccine access. Household wealth demonstrated persistent socioeconomic gradients across all quintiles, with children from the second through the highest wealth categories showing 25% to 156% higher odds of vaccination compared to the poorest quintile, with the middle wealth quintile exhibiting the highest adjusted odds (AOR = 2.56, 95% CI: 2.15–3.03, *p* < 0.001). Maternal education maintained significant independent effects at the primary (AOR = 1.58, 95% CI: 1.34–1.87, *p* < 0.001) and secondary (AOR = 1.94, 95% CI: 1.40–2.67, *p* < 0.001) levels, although the effect of higher education attenuated to non-significance after adjustment (AOR = 1.22, 95% CI: 0.73–2.03, *p* = 0.441), which should be interpreted cautiously given the small number of mothers with higher education in the sample. Residence type showed that nomadic populations retained 69% higher odds of vaccination (AOR = 1.69, 95% CI: 1.46–1.96, *p* < 0.001) than rural residents, while urban residence demonstrated a modest but significant association (AOR = 1.17, 95% CI: 1.01–1.36, *p* = 0.032), indicating that nomadic-targeted interventions may be more effective than previously assumed. Child age emerged as significant in the adjusted model, with children aged 12–23 months and 24–59 months showing 36% and 33% higher vaccination odds, respectively, compared to infants under 12 months (AOR = 1.36, 95% CI: 1.10–1.69 and AOR = 1.33, 95% CI: 1.12–1.59, respectively), potentially reflecting catch-up vaccination opportunities or age-related contact with healthcare services. Birth order remained independently associated with vaccination, with children of second or higher birth order having increased odds of vaccination compared with first-born children (AOR = 1.25, 95% CI: 1.13–1.40, *p* < 0.001). Media exposure effects were attenuated but remained significant, with a lack of radio exposure associated with 36% lower odds (AOR = 0.64, 95% CI: 0.50–0.82, *p* < 0.001) and the absence of television exposure showing marginal significance (AOR = 0.78, 95% CI: 0.61–1.00, *p* = 0.048). Regional disparities persisted substantially, with Togdheer (AOR = 2.45, 95% CI: 1.69–3.55), Sool (AOR = 2.35, 95% CI: 1.65–3.37), and Mudug (AOR = 1.83, 95% CI: 1.30–2.58) demonstrating significantly elevated odds, while the Gedo region exhibited the lowest odds (AOR = 0.26, 95% CI: 0.17–0.39, *p* < 0.001), reflecting profound geographic inequities potentially driven by conflict intensity, infrastructure availability, and program implementation effectiveness. Notably, maternal age and child sex remained non-significant predictors, even after adjustment, suggesting that these demographic factors do not independently influence vaccination uptake in the Somali context.

**Table 3:** Multivariable logistic regression of sociodemographic and healthcare factors associated with childhood vaccination among children aged 0–59 months, Somalia, 2020 Somalia Demographic and Health Survey (*n* = 7,373)

Variable	Category	Vaccinated *n* (%)	Not vaccinated *n* (%)	AOR (95% CI)	*P*-value
Mother’s age group	15–24 years	855 (33.1)	1,583 (32.9)	1	–
	25–34 years	1,251 (48.8)	2,256 (47.0)	1.06 (0.93–1.20)	0.379
	35–49 years	459 (18.1)	969 (20.1)	0.95 (0.81–1.11)	0.505
Child age group	0–11 months	265 (10.3)	558 (11.6)	1	–
	12–23 months	338 (13.2)	563 (11.8)	1.36 (1.10–1.69)	0.005
	24–59 months	1,962 (76.5)	3,687 (76.5)	1.33 (1.12–1.59)	0.001
Birth order	1st birth	1,450 (33.0)	2,944 (67.0)	1	—
	≥ 2 births	1,115 (37.4)	1,864 (62.6)	1.25 (1.13–1.40)	< 0.001
Place of delivery	Home delivery	1,775 (69.2)	4,167 (86.7)	1	–
	Health facility	790 (30.8)	641 (13.3)	1.93 (1.68–2.22)	< 0.001
Residence	Rural	619 (24.1)	1,383 (28.8)	1	–
	Urban	1,019 (39.7)	2,162 (45.0)	1.17 (1.01–1.36)	0.032
	Nomadic	927 (36.1)	1,263 (26.3)	1.69 (1.46–1.96)	< 0.001
Mother’s education	No education	1,925 (75.1)	4,254 (88.5)	1	–
	Primary	471 (18.4)	444 (9.3)	1.58 (1.34–1.87)	< 0.001
	Secondary	131 (5.1)	84 (1.7)	1.94 (1.40–2.67)	< 0.001
	Higher	38 (1.5)	26 (0.5)	1.22 (0.73–2.03)	0.441
Radio exposure	≥ once / week	223 (8.7)	183 (3.8)	1	–
	< once / week	107 (4.2)	96 (2.0)	1.06 (0.72–1.55)	0.769
	Not at all	2,235 (87.1)	4,529 (94.2)	0.64 (0.50–0.82)	< 0.001
TV exposure	≥ once / week	285 (11.1)	160 (3.3)	1	–
	< once / week	85 (3.3)	80 (1.6)	0.74 (0.50–1.11)	0.146
	Not at all	2,195 (85.6)	4,568 (95.0)	0.78 (0.61–1.00)	0.048
Wealth index	Lowest	386 (15.0)	1,397 (29.1)	1	–
	Second	400 (15.6)	1,344 (28.0)	1.25 (1.05–1.49)	0.012
	Middle	653 (25.5)	890 (18.5)	2.56 (2.15–3.03)	< 0.001
	Fourth	579 (22.6)	716 (14.9)	2.33 (1.95–2.79)	< 0.001
	Highest	547 (21.3)	461 (9.6)	2.45 (2.00–3.00)	< 0.001
Child sex	Male	1,353 (52.8)	2,479 (51.6)	1	–
	Female	1,212 (47.2)	2,329 (48.4)	0.98 (0.88–1.09)	0.709
Region	Awdal	85 (35.6)	154 (64.4)	1	
	Woqooyi Galbeed	143 (5.6)	257 (5.4)	1.20 (0.84–1.73)	0.322
	Togdheer	194 (7.6)	131 (2.7)	2.45 (1.69–3.55)	< 0.001
	Sool	228 (8.9)	166 (3.5)	2.35 (1.65–3.37)	< 0.001
	Sanaag	231 (9.0)	279 (5.8)	1.31 (0.93–1.86)	0.124
	Bari	177 (6.9)	294 (6.1)	1.17 (0.83–1.66)	0.368
	Nugaal	195 (7.6)	303 (6.3)	1.27 (0.90–1.80)	0.168
	Mudug	233 (9.1)	273 (5.7)	1.83 (1.30–2.58)	0.001
	Galgaduud	139 (5.4)	328 (6.8)	0.83 (0.58–1.19)	0.309
	Hiraan	110 (4.3)	280 (5.8)	0.68 (0.47–0.98)	0.038
	Middle Shabelle	153 (5.9)	282 (5.9)	0.88 (0.61–1.25)	0.469
	Banadir	236 (9.2)	694 (14.4)	0.55 (0.39–0.78)	0.001
	Bay	82 (3.2)	113 (2.4)	1.01 (0.66–1.55)	0.964
	Bakool	122 (4.8)	421 (8.8)	0.43 (0.30–0.62)	< 0.001
	Gedo	52 (2.0)	458 (9.5)	0.26 (0.17–0.39)	< 0.001
	Lower Juba	185 (7.2)	375 (7.8)	0.72 (0.51–1.02)	0.062

## Discussion

This study examined the determinants of childhood immunization coverage among children aged 0–59 months in Somalia, using nationally representative data from the 2020 Demographic and Health Survey. The analysis revealed an alarmingly low overall vaccination coverage of 34.8%, with approximately 65% of children remaining unvaccinated or incompletely vaccinated. Multivariable logistic regression identified several significant independent predictors of immunization uptake, including the place of delivery at health facilities, household wealth, maternal education, residence type, child age, birth order, media exposure, and geographic region. Conversely, maternal age and child sex were not significantly associated with vaccination status. These findings underscore the profound multifaceted nature of vaccination inequities in Somalia, where intersecting socioeconomic, healthcare access, maternal literacy, and geographical factors create substantial barriers to achieving equitable immunization coverage in this fragile and conflict-affected setting.

The identification of health facility delivery as the strongest predictor of childhood vaccination is a critical finding, consistent with evidence from neighboring East African countries [32, 33]. Children born in health facilities demonstrated nearly double the odds of vaccination compared with home deliveries, even after controlling for confounding variables [32]. This pattern aligns closely with studies from Ethiopia, where facility delivery was associated with more than three-fold higher odds of complete immunization coverage [34]. The mechanism underlying this association involves multiple pathways: facility births provide opportunities for immediate postnatal care contact; establishment of maternal health records that facilitate follow-up visits; exposure to health education about immunization schedules; and integration within the formal healthcare system that ensures continuity of care [35]. In Somalia’s context, where approximately 80% of deliveries occur at home, this finding highlights a critical entry point for strengthening vaccination programs [36]. The integration of immunization services with maternal and child health platforms, particularly through antenatal care and skilled birth attendance, has emerged as an evidence-based strategy for improving vaccine uptake [35, 37]. Health facility delivery was strongly associated with childhood vaccination (AOR = 1.93; 95% CI: 1.68–2.22), with children born in health facilities demonstrating nearly double the odds of vaccination compared with home deliveries. This association may reflect greater contact with health services around the time of birth. However, the cross-sectional design precludes causal conclusions, and the specific mechanisms linking place of delivery to vaccination outcomes were not examined in this study.​.

The persistent and substantial wealth gradient observed in this study, with children from the highest wealth quintile showing 2.45 times higher odds of vaccination compared to the poorest quintile, reflects deeply entrenched socioeconomic inequities in healthcare access [38]. This finding resonates with broader regional evidence from sub-Saharan Africa, where wealth-related disparities in immunization coverage have been consistently documented across multiple countries including Ethiopia, Kenya, and Uganda [39–41]. The wealth effect operates through multiple mechanisms, including geographic proximity to health facilities, the ability to afford transportation costs, reduced opportunity costs of time spent seeking services, better nutrition and child health status, and enhanced social capital facilitating healthcare navigation [36, 42]. A clear wealth gradient was observed, with children from higher household wealth quintiles showing increased odds of vaccination (Second: AOR = 1.25; Middle: AOR = 2.56; Fourth: AOR = 2.33; Highest: AOR = 2.45) compared with the poorest quintile. Although the middle-wealth quintile exhibited slightly higher adjusted odds, differences across the upper quintiles were modest and may reflect variability in the data rather than a distinct or systematic pattern. Addressing wealth-based inequities requires comprehensive policy responses, including the elimination of user fees for immunization services, provision of transportation vouchers or mobile vaccination services for remote populations, conditional cash transfer programs incentivizing vaccine uptake, and community-based insurance schemes that reduce financial barriers [43]. Somalia’s partnership with Gavi, the Vaccine Alliance, provides a strategic opportunity to implement pro-poor financing mechanisms and demand-side interventions, specifically targeting the lowest wealth quintiles [44].​.

Maternal education emerged as a powerful independent determinant, with primary and secondary education levels significantly associated with higher vaccination odds, although the effect of tertiary education was attenuated to non-significance after adjustment [45]. This dose-response relationship has been extensively documented across sub-Saharan Africa and reflects the multifaceted influence of maternal education on child health-seeking behavior [12, 13]. The Andersen Health Behavior Model provides a useful framework for interpreting this finding, positioning maternal education as a predisposing factor that enhances health literacy, improves the comprehension of immunization benefits and schedules, strengthens maternal autonomy and decision-making power within households, and facilitates effective communication with healthcare providers [46, 47]. Maternal education was positively associated with childhood vaccination, with primary (AOR = 1.58) and secondary education (AOR = 1.94) showing higher odds compared with no formal education. The association for higher education was not statistically significant after adjustment (AOR = 1.22; *p* = 0.441). This finding should be interpreted cautiously due to the very small number of mothers with higher education in the sample (*n* = 64; 0.9%), which limits statistical power. This nuanced finding has important programmatic implications, suggesting that simply providing information may be insufficient without addressing the structural barriers [32]. Effective interventions should combine community health education campaigns delivered through women’s groups and religious institutions, adult literacy programs integrated with health messaging, peer education models utilizing successfully vaccinated mothers, and mass media campaigns in local languages addressing vaccine misconceptions [48]. Given that 83.8% of the mothers in this sample had no formal education, investment in girls’ education represents a long-term structural intervention with profound intergenerational health benefits extending beyond immunization [39].​.

The counterintuitive finding that nomadic populations demonstrated 69% higher odds of vaccination compared to rural residents, with urban residence showing only a modest association, challenges conventional assumptions about immunization access in mobile pastoralist communities [49]. This pattern diverges from the typical findings in Ethiopia and Kenya, where nomadic populations consistently show lower coverage [50]. The relatively better performance of Somalia’s nomadic populations may reflect the successful implementation of targeted strategies documented in recent literature, including establishment of permanent transit vaccination points at livestock markets and water sources, integration of human and animal vaccination campaigns leveraging veterinary service infrastructure, engagement with clan leaders and traditional authority structures, cross-border coordination mechanisms for populations moving between Somalia, Ethiopia, and Kenya, and deployment of mobile vaccination teams specifically tracking nomadic movements [51, 52]. These innovative approaches, although operationally complex and costly, demonstrate that reaching mobile populations is feasible with appropriate investment and cultural adaptation [52, 53]. However, the lower coverage in rural areas highlights the insufficient penetration of routine immunization services in sedentary populations, suggesting that rural health infrastructure development has lagged behind targeted nomadic interventions [45]. Scaling successful nomadic vaccination strategies while simultaneously strengthening routine services in rural areas requires balanced resource allocation and context-specific delivery [45].​.

### Strengths and limitations of the study

The strengths of this study include the utilization of nationally representative Demographic and Health Survey data to provide robust estimates across diverse population subgroups; a large sample size of 7,373 children ensuring adequate statistical power; comprehensive assessment of individual, household, and community-level determinants; and rigorous analytical methods incorporating complex survey design adjustments. However, several limitations of this study warrant further investigation. The cross-sectional design precludes causal inference, limiting conclusions to associations rather than definitive causal pathways. Vaccination status was partially reliant on maternal recall in the absence of vaccination cards, potentially introducing recall and reporting bias; however, validation studies suggest reasonable accuracy for recent vaccinations. The binary outcome variable of vaccination versus no vaccination, while reflecting overall immunization system contact, does not capture the completion of full vaccination schedules or the timeliness of vaccine doses. Additionally, unmeasured confounders, including healthcare quality, provider attitudes, vaccine stockout frequency, and community-level social capital, could not be assessed using available DHS variables.

### Recommendation

Future research should prioritize several key areas to advance the understanding of immunization determinants and to inform targeted interventions. Qualitative studies examining vaccine hesitancy, cultural beliefs, and demand-side barriers among zero-dose children’s families could provide critical insights into behavioral change interventions. Prospective cohort studies that track children from birth through the completion of vaccination schedules could elucidate dropout patterns and critical decision points. Geospatial analyses integrating health facility location, conflict intensity mapping, and population distribution would inform optimal vaccination post-positioning and mobile team routing. Implementation of science research evaluating innovative delivery strategies such as the health camp model, integrated human-animal vaccination campaigns, and community-based vaccination by trained traditional birth attendants would generate evidence on scalable approaches for reaching hard-to-reach populations. Finally, vaccine supply chain analyses examining cold chain capacity, stockout patterns, and logistics bottlenecks would address critical supply side constraints that limit service availability.

## Conclusion

This study provides nationally representative evidence on the determinants of childhood immunization coverage in Somalia, highlighting substantial inequities associated with socioeconomic status, maternal education, healthcare access, place of residence, and geographic region. Vaccination uptake was consistently higher among children born in health facilities, those from wealthier households, children of educated mothers, and older age groups, while marked disparities persisted across regions and among disadvantaged populations.

These findings underscore the importance of addressing demand-side and access-related barriers that limit routine immunization uptake, particularly among children from the poorest households, those born outside health facilities, mothers with limited formal education, and populations residing in regions with persistently low coverage. Strengthening strategies that promote maternal engagement with health services, improve access to information, and reduce socioeconomic barriers may contribute to more equitable immunization outcomes.

While this study did not assess health system capacity or supply-side constraints, the observed associations highlight priority population groups that may benefit most from targeted, equity-focused immunization efforts. Future research integrating household-level data with facility- and system-level information would be valuable to further elucidate the mechanisms underlying regional and socioeconomic disparities in vaccination coverage.

Overall, improving childhood immunization coverage in Somalia will require sustained attention to social and structural inequalities influencing access to preventive health services. Addressing these disparities is essential for advancing child survival and strengthening progress toward equitable maternal and child health outcomes nationwide.

## Data Availability

The datasets analyzed in this study are available from the Demographic and Health Surveys (DHS) Program website ( [**https://dhsprogram.com**](https:/dhsprogram.com) ) upon reasonable request and registration. Researchers interested in accessing the 2020 Somalia DHS dataset can obtain it through the DHS Program’s standard registration and data request procedures.

## Abbreviations

AOR: Adjusted Odds Ratio
BCG: Bacillus Calmette-Guérin
CI: Confidence Interval
cOR: Crude Odds Ratio
COVID: 19-Coronavirus Disease 2019
DHS: Demographic and Health Survey
DPT: Diphtheria-Pertussis-Tetanus
IDP: Internally Displaced Persons
OPV: Oral Polio Vaccine
SDHS: Somalia Demographic and Health Survey
SDG: Sustainable Development Goal
UNICEF: United Nations Children’s Fund
WHO: World Health Organization
